# Sex-specific microRNAs in women with diabetes and left ventricular diastolic dysfunction or HFpEF associate with microvascular injury

**DOI:** 10.1038/s41598-020-70848-8

**Published:** 2020-08-18

**Authors:** Barend W. Florijn, Gideon B. Valstar, Jacques M. G. J. Duijs, Roxana Menken, Maarten J. Cramer, Arco J. Teske, Chahinda Ghossein-Doha, Frans H. Rutten, Marc E. A. Spaanderman, Hester M. den Ruijter, Roel Bijkerk, Anton Jan van Zonneveld

**Affiliations:** 1 Department of Internal Medicine (Nephrology), Leiden University Medical Center, Leiden, The Netherlands; 2 Einthoven Laboratory for Vascular and Regenerative Medicine, Leiden University Medical Center, Leiden, The Netherlands; 3 Julius Center for Health Sciences and Primary Care, University Medical Center Utrecht, Utrecht University, Utrecht, The Netherlands; 4 Division of Heart and Lungs, Department of Cardiology, University Medical Center Utrecht, University of Utrecht, Utrecht, The Netherlands; 5 Department of Obstetrics and Gynecology, Research School GROW, Maastricht University Medical Center, Maastricht, The Netherlands

**Keywords:** Biomarkers, Cardiology

## Abstract

Left ventricular diastolic dysfunction (LVDD) and heart failure with preserved ejection fraction (HFpEF) are microcirculation defects following diabetes mellitus (DM). Unrecognized HFpEF is more prevalent in women with diabetes compared to men with diabetes and therefore sex-specific diagnostic strategies are needed. Previously, we demonstrated altered plasma miRs in DM patients with microvascular injury [defined by elevated plasma Angiopoietin-2 (Ang-2) levels]. This study hypothesized the presence of sex-differences in plasma miRs and Ang-2 in diabetic (female) patients with LVDD or HFpEF. After a pilot study, we assessed 16 plasma miRs in patients with LVDD (n = 122), controls (n = 244) and female diabetic patients (n = 10). Subsequently, among these miRs we selected and measured plasma miR-34a, -224 and -452 in diabetic HFpEF patients (n = 53) and controls (n = 52). In LVDD patients, miR-34a associated with Ang-2 levels (R^2^ 0.04, R = 0.21, *p* = 0.001, 95% CI 0.103–0.312), with plasma levels being diminished in patients with DM, while women with an eGFR < 60 ml/min and LVDD had lower levels of miR-34a, -224 and -452 compared to women without an eGFR < 60 ml/min without LVDD. In diabetic HFpEF women (n = 28), plasma Ang-2 levels and the X-chromosome located miR-224/452 cluster increased compared to men. We conclude that plasma miR-34a, -224 and -452 display an association with the microvascular injury marker Ang-2 and are particularly targeted to women with LVDD or HFpEF.

## Introduction

Heart failure with preserved ejection fraction (HFpEF), and its precursor left ventricular diastolic dysfunction (LVDD), are manifestations of microvascular injury in diabetes patients^1^. The pathophysiology of microvascular injury in this patient population is associated with more hospitalization and cardiovascular death^2^. Especially women with type 2 diabetes mellitus (DM) have a higher prevalence of HFpEF compared to men (28% vs. 18.4%), which often remains undiagnosed until the later stage of the disease^3–7^. Therefore, the need to develop appropriate diagnostic strategies specific for women is critical, whereby the assessment of plasma microRNA (miR) levels could improve the detection of HFpEF and its precursor LVDD following type 2 DM.

Previously, we demonstrated that a select subset of circulating angiogenic miRs (among others miR-126, miR-130b, miR-223 and miR-660) are increased in plasma derived from diabetic nephropathy patients (both women and men) and associate with microvascular injury, as defined by elevated plasma levels of Ang-2 levels^8^. In addition, we have demonstrated in a review of the literature that the differential expression of (plasma) miRs between women and men involves at least two potential mechanisms: (1) double dosage of X-chromosome located (X-linked) miRs due to incomplete X-chromosome inactivation and (2) estrogen regulation of miR transcription and processing^9^. The augmented expression of endothelial X-linked miRs has been established to instigate microcirculation defects^9^ while higher plasma levels of these miRs have previously been shown to identify microvascular injury in women with other cardiovascular disease phenotypes such as idiopathic atrial fibrillation (iAF)^10^.

**Figure 1:**
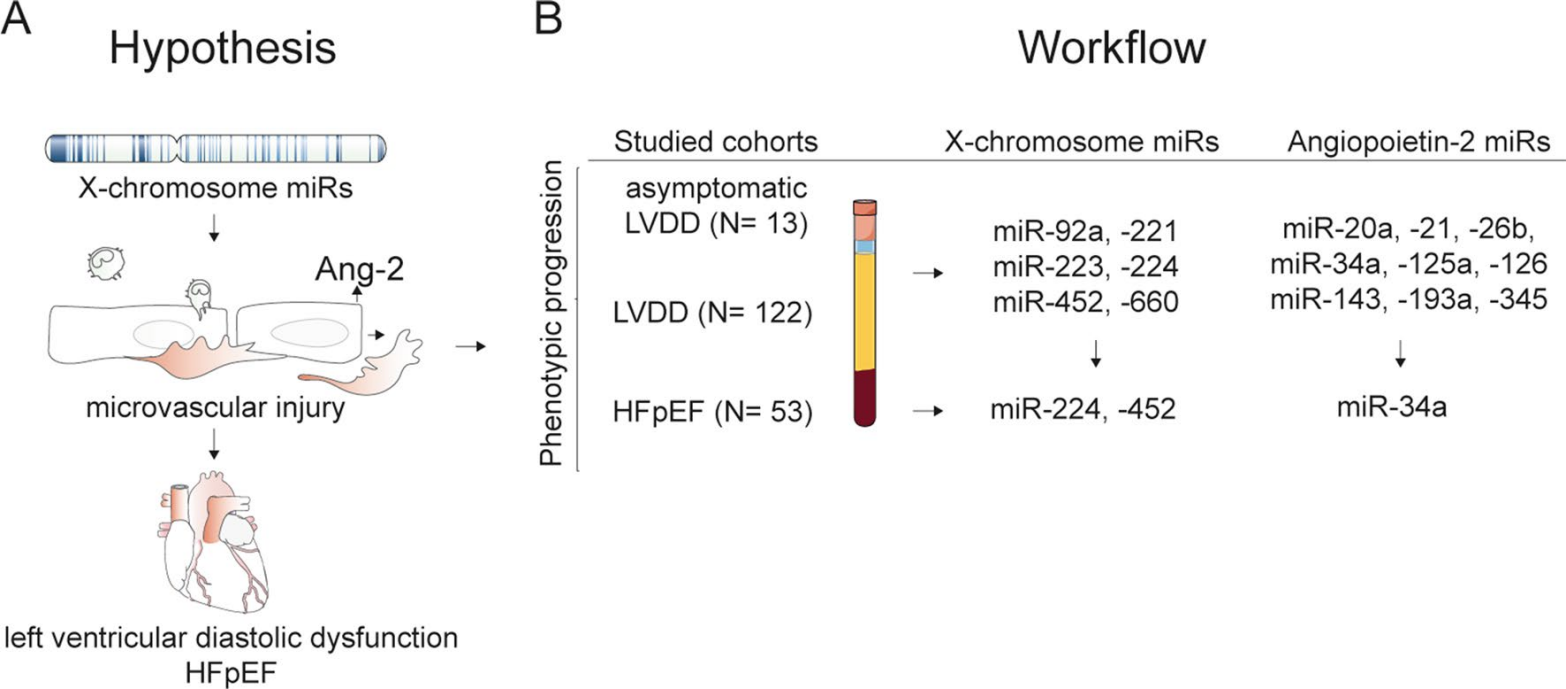
Hypothesis and workflow of the study. (**A**) This study hypothesized that sex-specific plasma miRs (derived from the X-chromosome) are differentially expressed in (female) patients with LVDD and HFpEF and associate with microvascular injury (defined by increased plasma Ang-2 levels). (**B**) In the workflow of the study we measured plasma Angiopoietin-2, X-chromosome located miRs and miRs that were previously found to associate with Ang-2 (Ref.^8,10,12^) in plasma from female patients with asymptomatic LVDD (n = 13). Next, we measured these miRs in patients with LVDD (n = 122) compared to their controls (n = 244). Subsequently and following analysis of the results we selected miR-34a. -224 and -452 and measured their plasma levels in patients with HFpEF (n = 52) compared to their respective controls (n = 53).

The present study hypothesizes that sex-specific plasma miRs are differentially expressed in (female) patients with HFpEF and its precursor LVDD (in particular in patients with diabetes) and associate with microvascular injury (defined by elevated plasma Ang-2 levels) (Fig. 1A). To test this, we assessed plasma Ang-2 (a tyrosine-protein kinase (TIE-2) receptor ligand that promotes endothelial activation and destabilization) and 16 candidate X-chromosome located (X-linked) miRs in (diabetic) LVDD and (diabetic) HFpEF patients. In addition, we performed sex-specific stratification of plasma- Ang-2 and miR level results and logistic regression analysis to assess which clinical characteristics are associated with significant differentially expressed plasma miRs.

## Results

### Patient characteristics

#### Asymptomatic LVDD patients (Maastricht study)

Supplemental Table 1 displays the clinical characteristics of patients with a previous history of preeclampsia and asymptomatic LVDD (n = 13) compared to healthy controls (n = 14). Women with asymptomatic LVDD had a higher ratio of mitral peak velocity of early filling (E) to early diastolic mitral annular velocity (E') (E/E' ratio) and a higher left atrial volume index (LAVI) compared to healthy parous controls.

#### LVDD patients (HELPFul study)

Table 1 displays the clinical characteristics of diabetic patients with LVDD (n = 12), derived from the HELPFul cohort, consisting of patients with LVDD (Supplemental Table 2). The HELPFul cohort is an ongoing case-cohort study at a Dutch cardiology outpatient clinic enrolling patients aged 45 years and older with no prior history of cardiovascular disease, who were referred by the general practitioner for cardiac evaluation^11^. Women with LVDD (n = 10) had a higher systolic- and diastolic blood pressure compared to women without LVDD (Table 1).

**Table 1:** (Sex-stratified) clinical characteristics of diabetes mellitus (DM) patients with or without LVDD.

	Total population		Women		Men	
	DM without LVDD (N = 17)	DM with LVDD (N = 12)	DM without LVDD (N = 7)	DM With LVDD (N = 10)	DM without LVDD (N = 10)	DM with LVDD (N = 2)
Sex, male, n (%)	10 (59%)	2 (17%)^1^	0 (0%)	0 (0%)		
Age (years)	63.1 ± 8.7	66.2 ± 8.5	63.1 ± 6.2	66.2 (8.9)	61.5 ± 10	68.4 ± 9.9
BMI (kg/m^2^)	27.8 ± 3.3	32.3 ± 7.0^1^	28.9 ± 3.7	33.6 ± 6.9	26.9 ± 3.9	27.1 ± 3.4
Systolic BP (mm Hg)	149.1 ± 15.9	156.7 ± 16.3	153.3 ± 16.3	156.5 ± 14.5	147.4 ± 19.6	158.1 ± 16.4
Diastolic BP (mm Hg)	85.3 ± 12.8	87.9 ± 9.2	85.8 ± 5.8	88.0 ± 9.5	88.1 ± 10.9	93.0 ± 10.7
Current or former smoker, n (%)	11 (65%)	8 (67%)	4 (57%)	6 (60%)	55 (65%)	27 (73%)
Hypertension, n (%)	13 (76%)	11 (92%)	6 (86%)	9 (90%)	48 (58%)	23 (64%)
Diabetes mellitus, n (%)	17 (100%)	12 (100%)	7 (100%)	10 (100%)	10 (100%)	2 (100%)
eGFR < 60 ml/min	2 (12%)	3 (25%)	1 (14%)	2 (20%)	1 (10%)	1 (50%)
Creatinine (umol/L)	68.8 (63.2–79.4)	67.6 (60.8–76)	68.1 (64.8–79.8)	63.8 (59.4–69.2)	83.2 (68.3–91.9)	95.1 (93.8–95.1)
Total cholesterol (mmol/L)	4.5 ± 0.9	4 ± 1.1	4.7 ± 0.7	4.0 ± 1.2	4.4 ± 1.1	3.8 ± 0.6
HDL-cholesterol (mmol/L)	1.3 ± 0.3	1.2 ± 0.2	1.5 ± 0.4	1.2 ± 0.3	1.1 ± 0.1	1.1 ± 0.2
Triglycerides (mmol/L)	2.2 ± 1.0	1.7 ± 1.1	2.6 ± 1.2	1.8 ± 1.2	1.8 ± 0.8	1.3 ± 0.07
**Echocardiography**						
Ejection fraction	66.8 (62.9–76)	68.4 (63.5–73.7)	66.8 (56.7–77.6)	68.4 (63.8–75.9)	66 (63.4–74.2)	66 (61.5–66.6)
EA ratio	0.8 ± 0.2	0.7 ± 0.1	0.9 ± 0.1	0.7 ± 0.1^1^	0.8 ± 0.3	0.7 ± 0.1
E/E’ ratio	8.9 (8.3–10.9)	9.4 (8.3–11.7)	9.6 (8.2–11.5)	10 (8.9–12.2)	8.8 (7.9–10.9)	8.2 (7.6–8.2)
LAVI (mL/m^2^)	23.7 (16.7–25.4)	25.6 (16–33.1)	24.3 (13.5–27.0)	24.3 (15.8–35.5)	22.9 (16.8–24.3)	28 (26.6–28) ^1^
LVMI (g/m^2^)	70.0 ± 15.6	82 ± 22.0	67 ± 9	78.3 ± 20.5	72 ± 19	100.5 ± 26.8
RWT	0.38 (0.31–0.47)	0.46 (0.41–0.48)	0.37 (0.31–0.39)	0.46 (0.42–0.47) ^1^	0.45 (0.30–0.52)	0.46 (0.41–0.46)
BNP (pg/mL)	16.9 (5–23.4)	11.5 (2.5–35.9)	24.8 (0–47.9)	11.4 (0–34.2)	13.4 (7.5–21.2)	42.5 (10.2–42.5)
e’ septal velocity (cm/sec)	7 (5–8.5)	6 (5–7)	8 (6–9)	6.5 (5–7.3)	6.5 (5–8.3)	5 (4–5)
e’ lateral velocity (cm/sec)	9 (7–10)	8 (6.3–9)	10 (9–10)	8 (5.8–8.3) ^1^	8 (6.8–9.5)	9.5 (9–9.5)

EA ratio, ratio of early (e) to late (a) ventricular filling; E/E’ ratio, ratio of mitral peak velocity of early filling (E) to early diastolic mitral annular velocity (E'); LAVI, left atrial volume index; LVMI, left ventricular mass index; RWT, relative wall thickness; BNP, brain natriuretic peptide; TPV, tricuspid valve regurgitation velocity. Parametric data is presented as mean ± SD or median ± SD. Non-parametric data is presented as median and IQR. Categorical data is presented as frequency and percentage. ^1^*p* < 0.05 versus no LVDD as determined by Independent samples t-test for all normally distributed continuous variables, Pearson chi-square test for binary variables and Independent Samples Mann Whitney U test for non-normally distributed variables.

#### HFpEF patients (UHFO-DM study)

Table 2 displays the clinical characteristics of DM patients with HFpEF (n = 53). HFpEF women (n = 28) had a higher E/e’ ratio than HFpEF men (n = 27), while HFpEF men had a higher LAVI and higher plasma levels of circulating brain natriuretic peptides (BNP).

**Table 2:** (Sex-stratified) clinical characteristics of diabetes patients with (N = 53) or without HFpEF (N = 52).

	Total population		Women		Men	
	Controls (N = 52)	HFpEF (N = 53)	Controls (N = 26)	HFpEF (N = 28)	Controls (N = 24)	HFpEF (N = 27)
Age (years)	69.8 ± 6.9	75.4 ± 6.8^1^	69.2 ± 7.5	75.2 ± 5.9^1^	70.4 ± 6.3	75.6 ± 7.7^1^
BMI (kg/m^2^)	27.6 ± 5.1	30.1 ± 4.1^1^	28.3 ± 6.0	31.5 ± 4.4^1^	26.8 ± 4	28.7 ± 3.3
Systolic BP (mm Hg)	159.7 ± 21.7	162.3 ± 22.0	165.6 ± 24.8	162.7 ± 21.7	153.8 ± 16.5	162.7 ± 22.8
Diastolic BP (mm Hg)	89 ± 11	89.9 ± 11.1	92 ± 12.3	91.3 ± 11.6	86 ± 8.6	88.4 ± 10.5
Current or former smoker	5 (8%)	9 (15%)	3 (10%)	2 (7%)	2 (7%)	7 (23%)
Hypertension	38 (63%)	44 (73%)	23 (77%)	26 (87%)	15 (50%)	18 (60%)
eGFR < 60 ml/min	7 (13%)	12 (22%)	5 (17%)	5 (19%)	2 (8%)	7 (25%)
Creatinine (umol/L)	77 (65–87)	77 (65–91)	70 (60.5–81)	67.5 (60.5–76.5)	82 (73.8–92.3)	89 (76–102)
Hypercholesterolemia	45 (75%)	38 (63%)	22 (73%)	20 (67%)	23 (77%)	18 (60%)
**Echocardiography**						
Ejection fraction	60.6 (54.5–65.9)	60.8 (55.0–65.6)	61.4 (55.8–66.6)	61.0 (56.9–68.2)	60.4 (54.2–63.0)	59.3 (53.4–64.9)
EA ratio	0.80 ± 0.19	0.83 ± 0.62	0.80 ± 0.19	0.80 ± 0.38	0.79 ± 0.19	0.87 ± 0.81
E/E’ ratio	8.6 (7.2–10.2)	10.6 (9.2–12.7)^2^	9.8 (8.0–10.8)	11.5 (10.3–14.7)^2^	8.3 (6.3–9.0)	9.9 (7.8–11)^2^
LAVI (mL/m^2^)	24.2 (18.4–29.9)	29.3 (25–34.1)^2^	24.4 (18–31.3)	27.7 (24.5–32.2)	24.0 (19.6–29.6)	32.4 (25.5–37.4)^2^
RWT	0.31 (0.28–0.34)	0.31 (0.26–0.38)	0.32 (0.28–0.34)	0.33 (0.30–0.41)	0.31 (0.27–0.24)	0.29 (0.24–0.36)
BNP (pg/mL)	8 (5–12)	13.5 (8–28.3)^2^	10 (5–16.5)	13 (8–22.5)	7 (4.8–9.3)	15.5 (8–37.3)^2^

EA ratio, ratio of early (e) to late (a) ventricular filling; E/E’ ratio, ratio of mitral peak velocity of early filling (E) to early diastolic mitral annular velocity (E'); LAVI, left atrial volume index; RWT, relative wall thickness; BNP, brain natriuretic peptide. Parametric data is presented as mean ± SD or median ± SD. Non-parametric data is presented as median and IQR. Categorical data is presented as frequency and percentage. ^1^*p* < 0.05 (significant difference between groups) as determined by Independent samples T-test for normally distributed continuous variables. ^2^*p* < 0.05 (significant difference between groups) as determined by Independent Samples Mann–Whitney U test.

### Pilot study identifies candidate miRs that associate with microvascular injury

We performed a pilot study to select candidate plasma miRs for plasma profiling in LVDD and HFpEF patients. In this study we assessed plasma levels of (1) 118 X-chromosome located miRs in healthy women and men (n = 6) (Supplemental Table 3). Out of differentially expressed miRs, we selected the X-linked miR-660 for further analysis in asymptomatic LVDD and LVDD patients (Fig. 1B).

Furthermore, based on our previously published studies into the association of plasma miRs with microvascular injury in diabetic nephropathy^8^, idiopathic atrial fibrillation^10^ and kidney transplant rejection^12^ as well as a review of the literature of miRs associated with angiogenesis, or their X-linked origin, we selected the following miRs for analysis in the second pilot study: miR-20a, miR-21, miR-26b, miR-34a, miR-92a, miR-125a, miR-126, miR-143, miR-193a, miR-221, miR-223, miR-224, miR-345, miR-452 (References can be found in Supplemental Table 4).

In the second pilot study, the aforementioned miRs were measured in plasma from women with a previous history of pre-eclampsia and asymptomatic LVDD (n = 13, Clinical characteristics in Supplemental Table 1) compared to healthy controls (n = 14) to determine a potential association with diastolic dysfunction. We observed that of the measured miRs, only plasma levels of miR-224 (FC 1.5, *p* = 0.03, 95% CI 0.095–0.598) were increased in women with asymptomatic LVDD (Supplemental Table 5 and Supplemental Figure 1A) compared to healthy parous controls.

Next, given that women with a history of preeclampsia have an increased risk for microvascular injury and heart failure^13^, we assessed whether women with preeclampsia and asymptomatic LVDD indeed display an association with microvascular injury. To that extent we measured markers of microvascular injury in plasma from women with a previous history of pre-eclampsia and asymptomatic LVDD (n = 13, Supplemental Table 1) As compared to healthy controls (n = 14), our assessment of Ang-2, soluble trombomodulin (sTM) and soluble fms-like tyrosine kinase-1 (sFlt-1) revealed that only plasma Ang-2 levels were higher in women with a history of preeclampsia and asymptomatic LVDD (fold change (FC) 1.4, *p* = 0.02, 95% CI 230.5–1,084) (Supplemental Table 5 and Supplemental Figure 1B).

Finally, we assessed the association between markers of microvascular injury (Ang-2, sTM, sFflt-1) and plasma miRs and observed moderate positive associations for Ang-2 and miR-34a (R = 0.36, FDR adjusted *p* = 0.02), -224 (R = 0.32, FDR adjusted *p* = 0.03), and miR-452 (R = 0.39, FDR adjusted *p* = 0.01). Similar associations were found for sTM and miR-125a (R = − 0.38, FDR adjusted *p* = 0.01), -126 (R = − 0.30, FDR adjusted *p* = 0.04) and miR-143 (R = − 0.39, FDR adjusted *p* = 0.01) (Supplemental Figure 1C).

### Plasma miR-34a in patients with diabetes and LVDD

**Figure 2:**
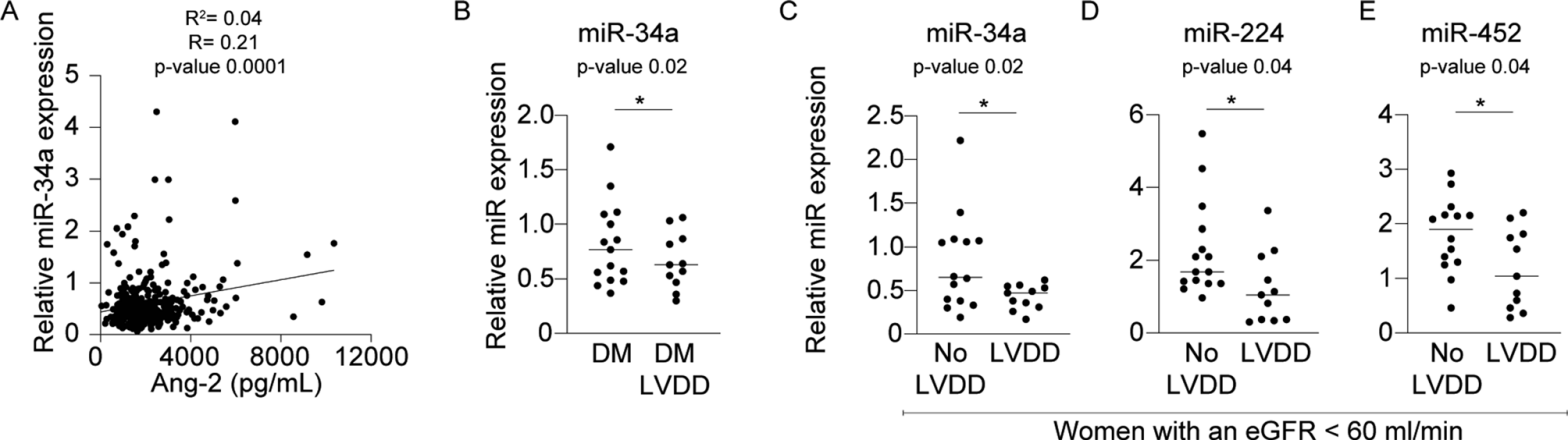
Plasma Ang-2 levels and circulating miR-34a, -224 and -452 in (female) DM patients with LVDD compared controls. (**A**) Particularly miR-34a displayed a significant correlation with Ang-2 in the total patient population of patients with LVDD (N = 366, R^2^ 0.04, R = 0.21, *p* = 0.001, 95% CI 0.103–0.312). (**B**) Lower levels of miR-34a in diabetic patients with LVDD compared to diabetic patients without LVDD (Fold change 1.2, *p* = 0.02, 95% CI − 0.428 to 0.117). (**C**) Lower levels of miR-34a, in women with an eGFR < 60 ml/min and LVDD compared to women without LVDD (Fold change 1.9, *p* = 0.03, CI − 0.737 to − 0.030). (**D**) Decreased plasma miR-224 in women with an eGFR < 60 ml/min and LVDD compared to women without LVDD (Fold change 1.8, *p* = 0.04, CI − 1.998 to − 0.059) and (**E**) decreased miR-452 in women with an eGFR < 60 ml/min and LVDD compared to women without LVDD (Fold change 1.5, *p* = 0.04, CI − 1.213 to − 0.039). Relative miR-expression values are normalized to plasma miR-16 levels, presented as mean ± standard error of the mean (SEM) and group differences are depicted as *p ≤ 0.05. Correlations between variables were calculated using the Spearman rank correlation.

Having identified a differential expression of plasma miR-224 and Ang-2 in women with asymptomatic LVDD, we next aimed to investigate whether patients with LVDD (Clinical characteristics in Supplemental Table 2) also have increased plasma miRs and Ang-2 levels. However, we observed that plasma miRs and Ang-2 were not significantly different in patients with LVDD (n = 122) compared to healthy controls (n = 244) (data not shown). Next, we assessed a potential association between plasma miRs and Ang-2 in patients with LVDD. Interestingly, in the entire cohort of patients with LVDD and controls we found that miR-34a was associated with plasma levels of Ang-2 (R^2^ 0.04, R = 0.21, *p* = 0.001, 95% CI 0.103–0.312, Fig. 2A).

**Figure 3:**
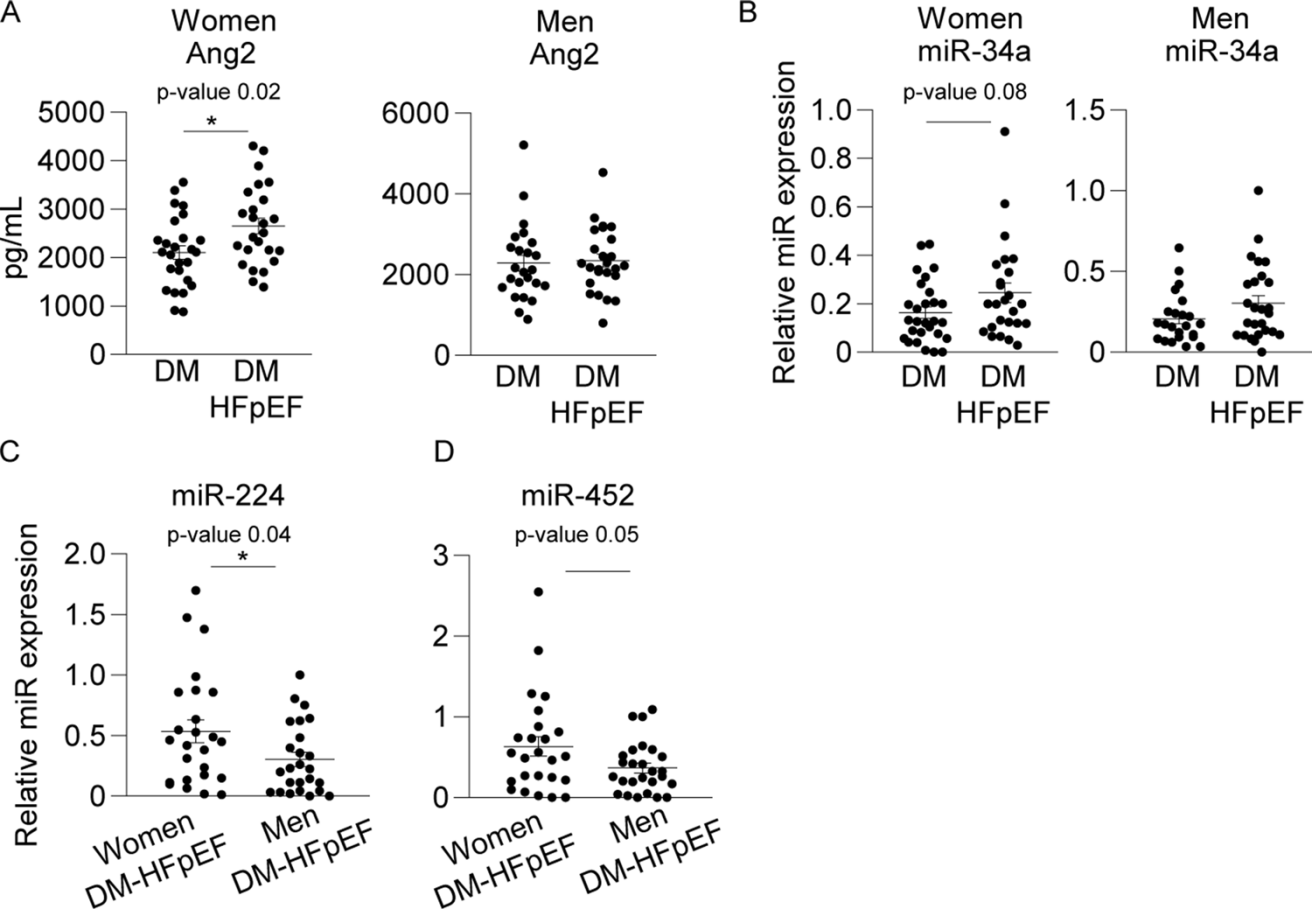
Plasma Ang-2 levels and plasma miR-34a, -224 and -452 in (female) DM patients with HFpEF. (**A**) Increased plasma Ang-2 (Fold change 1.3, *p* = 0.02, 95% CI 110.5–980.9) levels in diabetic women with HFpEF compared to diabetic women without HFpEF (but not in diabetic HFpEF men compared to diabetic men without HFpEF), (**B**) miR-34a displayed a trend towards an increase in diabetic women with HFpEF compared to diabetic women without HFpEF only (Fold change 1.5, *p* = 0.08, 95% CI − 0.009 to 0.173). (**C**) Increased plasma levels of miR-224 (Fold change 0.57, *p* = 0.04, 95% CI − 0.452 to − 0.010) and (**D**) miR-452 (Fold change 0.58, *p* = 0.05, − 0.539 to − 0.002) in diabetic women with HFpEF compared to diabetic men with HFpEF. Relative miR expression values are normalized to plasma miR-16 levels, presented as mean ± standard error of the mean (SEM) and group differences are depicted as *p ≤ 0.05 according to a student T-test.

Subsequently, we conducted a linear regression analysis to assess which clinical characteristics determine plasma miR-34a in patients with LVDD. This analysis (Table 3) identified that particularly sex (β = − 0.160, *p* value = 0.002) and the presence of DM (β = 0.121, *p* value = 0.02) were statistically significant determinants of this relationship while an eGFR < 60 ml/min displayed a trend towards an association, although non-significant (β = − 0.101, *p* value = 0.09). Given that the presence of DM was a significant determinant of plasma miR-34a, we assessed plasma levels of miR-34a in patients with diabetes, with or without LVDD (Table 1) and found significant lower plasma levels of miR-34a in diabetic patients with LVDD compared to diabetic patients without LVDD (FC 1.23, *p* = 0.02, 95% CI − 0.428 to 0.117, Fig. 2B).

**Table 3:** Multiple linear regression analysis of the association between clinical characteristics and plasma miRs.

	miR-34a		miR-224		miR-452	
	β	*p* value	β	*p* value	β	*p* value
Age	0.106	0.097	0.004	0.955	0.002	0.970
Sex	− 0.160	0.002	0.145	0.007	0.165	0.002
eGFR	− 0.101	0.099	− 0.035	0.579	− 0.101	0.107
Hypertension	− 0.098	0.065	− 0.045	0.412	− 0.046	0.402
Diabetes Mellitus	0.121	0.022	0.033	0.537	0.020	0.711
Obesity	0.113	0.035	0.010	0.851	− 0.074	0.173

In the linear regression model, miRs are dependent variables while age, sex, eGFR, hypertension, diabetes mellitus and obesity are independent variables. A simultaneous method of entry was used in the regression model. “β” means standardized regression coefficients; “*p* value” indicates significance.

### Sex-specific plasma miR-34a, -224 and -452 levels in women with an eGFR < 60 ml/min and LVDD

In our pilot study in women with asymptomatic LVDD we observed significant correlations between plasma Ang-2 and miR-224 and -452. Therefore, we used linear regression analysis (Table 3) to investigate if sex could determine plasma miR-34a, -224 and -452 levels in patients with LVDD. We found that indeed sex was a significant determinant of this relationship for miR-34a (β = − 0.160, *p* = 0.002), miR-224 (β = 0.145, *p* = 0.002) and miR-452 (β = 0.165, *p* = 0.007). Interestingly, linear regression analysis also demonstrated that an eGFR < 60 ml/min displayed a (non-significant) trend as determinant of plasma miR-34a (β = − 0.101, *p* = 0.09), while an eGFR < 60 ml/min could not determine plasma miR-224 (*p* = 0.58) or miR-452 (*p* = 0.11).

Subsequently, we assessed differential expression of these miRs in patients with an eGFR < 60 ml/min with or without LVDD (clinical characteristics in Supplemental Table 6. Results from this analysis demonstrated that in particular female patients with LVDD with an eGFR < 60 ml/min displayed lower levels of miR-34a (FC 1.9, *p* = 0.03, CI − 0.737 to − 0,030, Fig. 2C), miR-224 (FC 1.8, *p* = 0.04, CI − 1.998 to − 0.059, Fig. 2D) and miR-452 (FC 1.5, *p* = 0.04, CI − 1.213 to − 0.039, Fig. 2E) compared to female patients with an eGFR < 60 ml/min without LVDD.

### Plasma Ang-2 and miR-34a, -224 and -452 levels in women with diabetes and HFpEF

Next we hypothesized that the phenotypic progression from LVDD to HFpEF in the setting of DM could be associated with pronounced microvascular injury, and increased Ang-2 levels. For this, we measured plasma Ang-2 levels in DM patients with HFpEF (Table 2, n = 53). Here, we observed increased levels of plasma Ang-2 (FC 1.3, *p* = 0.02, 95% CI 110.5–980.9, Fig. 2A) in diabetic women with HFpEF (n = 28) as compared to diabetic women without HFpEF (n = 27).

Next, we selected miR-34a, -224 and -452 based on pilot study results and measured their plasma levels in DM patients with HFpEF compared to DM patients without HFpEF. In diabetic patients (both women and men) with HFpEF (n = 53) compared to diabetic patients without HFpEF (n = 52), we observed no significant differences in miR-224 and -452 levels (data not shown). However, plasma levels of miR-34a displayed a trend towards an increase in diabetic women with HFpEF (n = 28) compared to diabetic women without HFpEF (n = 27, FC 1.5, *p* = 0.08, 95% CI − 0.009 to 0.173, Fig. 2B). In addition, when women with HFpEF (n = 28) were compared to men with HFpEF (n = 27), the plasma levels of miR-224 (FC 0.57, *p* = 0.04, 95% CI − 0.452 to − 0.010, Fig. 2C) and miR-452 (FC 0.58, *p* = 0.05, − 0.539 to − 0.002, Fig. 2D) were significantly higher in women versus men with HFpEF.

## Discussion

In this study, our main findings were (1) decreased levels of miR-34a, -224 and -452 in diabetes patients with LVDD and in female diabetes patients with an eGFR < 60 ml/min but increased plasma miR-224 and miR-452 in diabetic women with HFpEF versus diabetic men with HFpEF; (2) regression analysis demonstrated that sex, DM and renal dysfunction are associated with plasma miR-34a levels in patients with LVDD; and (3) Increased levels of plasma Ang-2 in diabetic women with HFpEF but not in diabetic men with HFpEF.

MiRs are extensively involved in manifestations of microvascular injury following type 2 DM^14^. In the setting of HFpEF, the X-linked miR-545-5p differentiated HFpEF patients from healthy controls^15^ while X-linked miR-221 distinguished HFpEF patients from patients with heart failure with a reduced ejection fraction (HFrEF)^16^. Nonetheless, statistical analysis of these miR expression results were not stratified for women and men, and the identified miRs were not related to molecular pathways involved in microvascular injury^17^. In this study, plasma miR-34a levels were decreased in diabetic patients with LVDD, while miR-34a, -224 and -452 were decreased in diabetic women with LVDD and an eGFR < 60 ml/min. In contrast, miR-34a was increased in diabetic women with HFpEF (although non-significant) while miR-224 and -452 were increased in diabetic HFpEF women versus men. This dichotomous outcome in plasma miR-34a levels could indicate different pathophysiological mechanisms of plasma miR release in different phenotypes of diastolic dysfunction. This was previously demonstrated with plasma miRs following different stages of cardiomyocyte remodeling in patients with aortic stenosis^18^. It could also indicate that different stages of diastolic dysfunction change the distribution of circulating miRs among plasma carriers like high density lipoprotein (HDL), extracellular vesicles and plasma Argonaute-2 protein, like we previously demonstrated following a phenotypic progression from DM to diabetic nephropathy^19^. Therefore, instead of the overall absolute quantity of total plasma miRs, a specific difference in the distribution of miRs among these carriers could potentially provide a better explanation for this dichotomous trend and its relationship to microvascular injury in diastolic dysfunction. Still, regression analysis in this study identified that sex was found to be a statistically significant determinant of plasma miR-34a in LVDD patients. This could potentially explain the observed increase in women as compared to men with HFpEF and suggests a similar sex specific plasma expression pattern as was previously shown for miR-34a^20^. Nonetheless, it could also very well be that the small sample size of diabetic women with LVDD (n = 10) has led to his differential expression of plasma miRs. Therefore, additional well powered studies are needed to investigate sex differences in plasma miR trends in different stages of diastolic dysfunction.

In this study we observed that women with asymptomatic LVDD as well as diabetic women with HFpEF had higher plasma Ang-2 levels, a finding not present in diabetic women with LVDD. Again, this could be related to the relatively small cohort size of diabetic women with LVDD (n = 10). Still, higher levels of plasma Ang-2 in the total cohort of LVDD patients and controls (n = 366) were modestly associated with miR-34a which could indicate a possible relevance of this marker for microvascular injury in these patients with LVDD. Higher plasma Ang-2 levels, indicating an association with microvascular injury in HFpEF women, is consistent with studies that demonstrated that coronary microvascular dysfunction is a female specific pathophysiology in HFpEF^21,22^. In addition, significantly higher plasma levels of miR-224 and -452 were seen in HFpEF women compared to HFpEF men. It could very well be that the X-chromosome origin of both miRs is associated with a differential expression in women only. Previously we reviewed that particularly X-chromosome located miRs instigate microvascular injury phenotypes which could explain why both miRs were higher in HFpEF women together with higher levels of the microvascular injury marker Ang-2^9^. However additional validation studies are necessary to evaluate this potential.

Of the identified plasma miRs in this study, miR-34a is predominantly secreted by adipocytes and promotes a systemic inflammatory state^23^. Interestingly, this miR has a well-established myocardial function in women because a targeted myocardial therapy with miR-34a in murine models of myocardial hypertrophy was previously found to reduce cardiac fibrosis and improved cardiac contractility in female mice^24^. More evidence for sex-specific downstream effects of this particular miR comes from the fact that it regulates the expression of the long-noncoding RNA X-inactive-specific transcript (XIST), which regulates X-chromosome inactivation in female cells^25^. In down regulating the expression of XIST (which normally mediates X-chromosome inactivation), miR-34a could increase the expression of both miR-224 and -452 which are located on a chromosomal cluster on the X-chromosome (source miRBase version 22). Of the X-linked miR-224/452 cluster, which is also predominantly expressed in adipose tissue^26^, miR-224 was increased in our pilot study in women with asymptomatic LVDD (Supplemental Figure 1A) and in diabetic women with HFpEF versus HFpEF diabetic men, while both miRs were decreased in women with an eGFR < 60 ml/min and the presence of LVDD (Fig. 2D,E). Regarding miR-224, this miR is known for regulating adipocyte differentiation^27^, inflammation^28^, and (micro)vascular quiescence or activation^29^. Also miR-452 has a role in metabolism and inflammation because hyperglycemia regulates miR-452 expression^30^ and its adipocyte-specific decrease activates a TNF-α induced inflammatory response^31^. However, more studies are needed to investigate whether these miRs pinpoint adipocyte dysfunction as a co-contributor to microvascular injury in diabetic women with LVDD or HFpEF.

In this study we measured Ang-2 as marker of microvascular injury in patients with HFpEF and LVDD. However, Ang-2 is a single protein biomarker which may not be able to capture (microvascular) disease variability in patient populations. Therefore, additional studies are needed to assess additional parameters of endothelial and microvascular function to confirm the presence of microvascular injury over the phenotypic progression from LVDD to HFpEF. Alternatively, a comparative analysis of circulating non-coding RNAs and other protein biomarkers could potentially better capture the different molecular characteristics at the endothelial and cardiomyocyte level upon a systemic inflammation induced microvascular injury phenotype. An interesting approach for such a comparative analysis would be to measure the miR-224/452 cluster and Pentraxin-3, a miR-224 target, involved in the innate immune- and inflammatory response^32^, produced in the coronary microcirculation and altered upon left ventricular (LV) diastolic dysfunction in patients with HFpEF^33^. Such an approach might be further strengthened by adding the measurement of miR-34a, that regulates cardiac contractile function^34^, and its gene target Bcl-2 which is pro-fibrotic and known to be involved in the myocardial structural abnormalities of HFpEF as well^35^. This could clarify whether the identified plasma miRs in this study indeed pinpoint to adipocyte dysfunction as a pathophysiological substrate for endothelial dysfunction and microvascular injury in (female) patients HFpEF and LVDD.

## Materials and methods

### Ethics and approval

This study complied with the ethical principles of the Declaration of Helsinki and was conducted following approval by the Institutional Review Board of the Maastricht University Medical Center (MUMC), Utrecht University Medical Center (UMCU) and the Leiden University Medical Center (LUMC). All patients provided written informed consent.

### Study designs

#### Maastricht study

Women with a history of preeclampsia (PE) were evaluated routinely for cardiovascular function at approximately 1 year postpartum. For this study, those women who were at least 4 years postpartum were invited by mail to participate in a second follow-up cardiovascular assessment. PE was diagnosed according to the criteria set by the International Society of Hypertension in Pregnancy: new-onset hypertension, systolic blood pressure (SBP) ≥ 140 mmHg and/or diastolic blood pressure (DBP) ≥ 90 mmHg, after 20 weeks’ gestation and proteinuria exceeding 0.3 g/day^36^.

#### HELPFul study

Study design and procedures of the “Discovery of biomarkers for the presence and progression of left ventricular diastolic dysfunction and HEart faiLure with Preserved ejection Fraction in patients at risk for cardiovascular disease” (HELPFul) study have been published in more detail^11^. Briefly, HELPFul is a Dutch case-cohort in which patients from the UMCU participated who were referred by their GP for a diagnostic cardiac assessment. Patients who had a previous cardiac intervention, or who were known with congenital cardiac disease were excluded from participation. Patients that had a ratio of the peak early (E) diastolic filling velocity and early diastolic mitral annular velocity (e′) (average of septal and lateral) (E/e′) ≥ 8 with tissue Doppler echocardiography were considered to have a higher probability of having LVDD. ‘Cohort’ patients were randomly sampled from all patients aged 45 years or older, striving to include 25% of eligible participants. Information on co-morbidities, medical history, and medication use was collected. The diagnostic work-up further consisted of physical examination, blood testing of standard cardiovascular biomarkers, electrocardiogram (ECG), bicycle exercise-ECG, and transthoracic echocardiogram. A structured case record form was used to assess symptoms suggestive of cardiac pathology. Hypertension was determined by (1) self-reporting, (2) use of blood pressure lowering medication, or (3) a mean (of at least two measurements) systolic blood pressure > 140 mmHg at the outpatient center. Type 2 diabetes was determined by self-reporting or use of blood glucose lowering medication. Hypercholesterolemia was determined by self-reporting or use of lipid-lowering medication. Atrial fibrillation was determined by self-reporting or atrial fibrillation on ECG at the outpatient center. Body mass index (BMI) was calculated from dividing weight (kg) by squared height in meters (m^2^). Waist to hip ratio was calculated from dividing waist circumference (cm) by hip circumference (cm). The estimated glomerular filtration rate (eGFR) was calculated from both creatinine and cystatin-c with the validated CKD-EPI formula^37^.

#### UHFO-DM study

UHFO-DM is a prospective diagnostic efficiency study from the UMCU^38^. Patients aged 60 years and over with diabetes type 2, enlisted with the diabetes service of the Center for Diagnostic Support in Primary Care (SHL), Etten-Leur were eligible. This was a representative sample of all patients with diabetes type 2 registered with a general practitioner. The standard care of the diabetes service consists of periodically serum glucose and HbA1c assessment and yearly monitoring of other laboratory parameters and fundoscopy, to help the general practitioners with the management of patients with diabetes. Furthermore, the SHL provides a supporting service to the general practitioners of diabetic nurses, who work according to the current diabetes guidelines. In total 561 general practitioners in the region make use of the services of the Diabetic service of the SHL, with 48,175 patients with type 2 diabetes enlisted in the SHL service. In total, 100 nurse practitioners from the SHL support more than 200 general practitioners (GPs) in their work for diabetic patients. A random sample of approximately 1,200 patients were enlisted within the SHL diabetic service database and when living within 60 km of the cardiology outpatient department of the Oosterschelde hospital in Goes they were asked to participate in the study. To prevent duplicate investigations, patients known with a cardiologist-confirmed diagnosis of heart failure, were only asked to fill out the questionnaires. They also were asked for permission to scrutinize their medical files for co-morbidities and date of diagnosis of heart failure.

### Echocardiography

Echocardiography measurements were carried out as previously described^13^ and performed in accordance with the recommendation of the American Society of Echocardiography (ASE)^39^. Echocardiographic measurements were made using a phased‐array echocardiographic Doppler system (Vivid 7, GE Vingmed Ultrasound, Horten, Norway). Left ventricular end‐diastolic (LVEDd), end‐systolic (LVESd) diameters (mm), end‐diastolic interventricular septum thickness (IVST; mm) and the posterior (inferolateral) wall thickness (PWT; mm) were measured using the M‐mode in the parasternal long‐axis view. Left ventricular mass (LVM; g) was determined using the formula 0.8 × (1.04((LVEDd + PWT + IVST)3 − (LVEDd)3)) + 0.6, indexed for body surface area^40^. Relative wall thickness (RWT) was computed using the formula (2 × PWT)/LVEDd)^40^. Left ventricular end‐diastolic (LVEDV, mL) and end‐systolic (LVESV, mL) volumes were determined using the Teichholz formula^41^. Heart rate (HR, bpm) was calculated by multiplying by 60 the reciprocal of the mean of five consecutive RR intervals on the electrocardiogram while ((LVEDV – LVESV)/(LVEDV)) × 100 was used to calculate left ventricular ejection fraction (LVEF, %). Stroke volume (SV, mL) was computed by taking the product of VTI and mid‐systolic cross‐sectional area (cm2) at the level of the left ventricular outflow tract in the parasternal long‐axis view. Mean aortic velocity time integral (VTI, cm) was calculated by averaging the outer edge tracings of five consecutive continuous wave Doppler registrations of the left ventricular outflow tract velocity. Cardiac output (CO, L/min) was calculated by multiplying SV by HR. The E/A ratio is the ratio of the early (E) to late (A) ventricular filling velocities while E/E’ is the ratio of mitral peak velocity of early filling (E) to early diastolic mitral annular velocity (E'). Using tissue doppler (TD), early (*e*') diastolic velocities were assessed at the septal and lateral insertion of the annulus of mitral valve. Assessments were executed offline using EchoPAC PC SW (GE Vingmed Ultrasound) version 6.1.2.

### Adjudication of diagnosis of asymptomatic LVDD (Maastricht study), LVDD (HELPFul study) and HFpEF (UHFO-DM study)

#### Maastricht study

Asymptomatic LVDD (heart failure stage B) was diagnosed according to the guidelines of the American Heart Association^42^. Asymptomatic LVDD was defined as the presence of previous myocardial infarction, LV hypertrophy (left ventricular mass index (LVMi) > 95 g/m^2^), concentric remodeling (RWT > 0.42 and LVMi ≤ 95 g/m^2^), mildly impaired LVEF (> 40% and < 55%) or asymptomatic valvular disease^40^ . We defined asymptomatic valvular disease as mild aortic valve insufficiency or central aortic valve insufficiency. HF with preserved ejection fraction (HFpEF) in this subclinical stage was defined as LVEF ≥ 55% but with the occurrence of one of the other criteria for asymptomatic LVDD^40^.

#### HELPFul study

We applied consensus diagnosis for LVDD with an expert panel consisting of cardiologists (RM, MJC, AT) and a general practitioner specialized in heart failure (FR). This method is comparable to previous studies^43,44^. The expert panel used all available diagnostic information, including patient reported symptoms, risk factors, electrocardiography, echocardiography, results from the exercise test, (cardiovascular) medication use and plasma B-type natriuretic (BNP) levels. The panel based the diagnosis of LVDD on available diastolic function criteria and recommended cut-points of recent international guidelines^45–47^. The panel categorized patients into four groups; no LVDD, possible LVDD, probable LVDD, and definite LVDD. For the purpose of this study, we combined probable LVDD with definite LVDD into ‘LVDD’, and no LVDD and possible LVDD into ‘no LVDD’.

#### UHFO-DM study

Presence or absence of HF was determined by an outcome panel consisting of two cardiologists and one GP^38^. The panel used all available information from the diagnostic work-up, including echocardiography, but except the NT-proBNP results (to prevent incorporation bias pertaining to this particular test). In case of no consensus the majority decision was used. For HFpEF patients had to have echocardiographic diastolic abnormalities in combination with indicative symptoms and signs (that is, peripheral or pulmonary fluid retention or raised jugular venous pressure) of heart failure or indicative symptoms and echocardiographic left ventricular hypertrophy, atrial fibrillation, or anginal complaints.

### RNA isolation

Plasma RNA was isolated from 200μL human plasma with 800μL Trizol reagent (Invitrogen, Breda, the Netherlands) using the RNeasy Micro Kit (Qiagen, 1082 Venlo, the Netherlands) as described previously^19^. Briefly, chloroform was added to the plasma/Trizol mixture and centrifuged for 15 min at 15,000 g. The aqueous phase was combined with 1.5 × the volume of 100% Ethanol, conveyed to a MinElute Spin column (Qiagen) and centrifuged for 15 s at 18.000*g*. Subsequently the RNA was washed with 700 μL RWT buffer, twice with 500 μL RPE buffer and centrifuged for 15 s at 18000*g* after the first two washing steps and 2 min at 18,000 g after the last washing step. Finally, RNA was eluted with 15 μL RNase-free water.

### MicroRNA profiling

For miR cDNA synthesis, reverse transcription of total RNA was performed using the miR reverse transcription kit (Applied Biosystems, Foster City, CA). cDNA was preamplified using Megaplex PreAmp primers pools according to the protocol of the manufacturer. For the pilot study custom designed megaplex cards were generated to determine the expression of 118 X-chromosome located miRs, 2 Y-chromosome located miRs, a selected set of 48 microvacular injury associated miRNAs^8,12^ and U6 and miR-16 as controls. Megaplex arrays were run and analyzed on a 7900HT Fast Real-Time PCR System (Applied Biosystems). In the main patient studies, expression of individual miRNAs was detected by dedicated TaqMan qRT-PCR assays as previously described^8^. After comparison of miR-16 en U6 Ct-values and their respective standard deviation (SD) in asymptomatic LVDD patients, LVDD patients and HFpEF patients, we choose to normalize miR expression results using expression levels of miR-16^13^.

### ELISA

In the Maastricht cohort, plasma soluble Flt-1 (sFlt-1), thrombomodulin (sTM) and Angiopoietin (Ang-2) concentrations were determined by ELISA (R&D System s, Minneapolis, MN and Diaclone Research, Besancon, France) according to the manufacturers’ supplied protocols. In the HELPFul and UHFO-DM cohort plasma Ang-2 concentration was determined by ELISA (R&D System s, Minneapolis, MN and Diaclone Research, Besancon, France) according to the manufacturers’ supplied protocols.

### Data analyses

Baseline characteristics are presented as mean ± standard deviation (SD), while non-normally distributed data is presented as median with interquartile range (IQR) and categorical data is presented as frequency with percentage. Differences between groups were compared using a Pearson Chi-square test or a student T-test, depending on the distribution of and type of variable. MiRNA-expression values are presented as mean ± standard error of the mean (SEM) and group differences were assessed using a student’s T-test. To account for the effect of age we also assessed differences of miRNA expression values between groups with one-way analysis of covariance (ANCOVA), adjusting for age. Correlations between variables were calculated using the Spearman rank correlation while *p* values were adjusted with multiple testing through FDR with Benjamini Hochberg correction. In the linear regression model, a simultaneous method of entry was used in which plasma miRs were selected as dependent variables while age, sex, eGFR, hypertension, diabetes mellitus and obesity as independent variables. A *p* value < 0.05 was considered to be statistically significant. Data analysis was performed using SPSS version 20.0 (SPSS, Inc., Chicago, IL) and GraphPad Prism, version 5.0 (GraphPad Prism Software, Inc., San Diego, CA).

## Supplementary information

Supplementary Information.

## Data Availability

The datasets generated and analyzed during the current study are available in the tables as provided in this manuscript. Any additional information on the data can be requested from the corresponding author.

## Abbreviations

MiR: MicroRNA
HFpEF: Heart failure with preserved ejection fraction
HFrEF: Heart failure with a reduced ejection fraction
LVDD: Left ventricular diastolic dysfunction
DM: Diabetes mellitus
X-linked: X-chromosome located
XIST: X-inactive-specific transcript
Ang-2: Angiopoietin-2
sFlt-1: Plasma soluble Flt-1
sTM: Plasma soluble thrombomodulin
PE: Preeclampsia
SBP: Systolic blood pressure
DBP: Diastolic blood pressure
GP: General practitioner
ECG: Electrocardiogram
BMI: Body mass index
GFR: Glomerular filtration rate
LVEDd: Left ventricular end‐diastolic diameter
LVESd: Left ventricular end‐systolic diameter
IVST: Interventricular septum thickness
PWT: Posterior (inferolateral) wall thickness
LVM: Left ventricular mass (LVM; g) was determined using the formula
RWT: Relative wall thickness
HR: Heart rate
CO: Cardiac output
TD: Tissue doppler
