# Supplementary material for: Sex-specific microRNAs in women with diabetes and left ventricular diastolic dysfunction or HFpEF associate with microvascular injury
Source: Sci Rep. 2020 Aug 18;10:13945. doi: 10.1038/s41598-020-70848-8 (PMC7435264; doi:10.1038/s41598-020-70848-8)
Supplement: Supplementary file 1 — Supplementary Information. [file 41598_2020_70848_MOESM1_ESM.docx]

**Supplemental Data**

**Sex-specific microRNAs in women with diabetes and left ventricular diastolic dysfunction or HFpEF associate with microvascular injury**

Barend W. Florijn^1,2^ ([b.w.florijn@lumc.nl](mailto:b.w.florijn@lumc.nl))

Gideon B. Valstar^3,4^ ([G.B.Valstar@umcutrecht.nl](mailto:G.B.Valstar@umcutrecht.nl))

Jacques M.G.J. Duijs^1,2^ ([J.M.G.J.Duijs@lumc.nl](mailto:J.M.G.J.Duijs@lumc.nl))

Roxana Menken^4^ ([r.menken@cardiologiecentra.nl](mailto:r.menken@cardiologiecentra.nl))

Maarten J. Cramer^4^ ([M.J.M.Cramer@umcutrecht.nl](mailto:M.J.M.Cramer@umcutrecht.nl))

Arco J. Teske^4^ ([A.J.Teske-2@umcutrecht.nl](mailto:A.J.Teske-2@umcutrecht.nl))

Chahinda Ghossein-Doha^5^ ([c.ghossein@maastrichtuniversity.nl](mailto:c.ghossein@maastrichtuniversity.nl))

Frans H. Rutten^3,4^ ([frutten@umcutrecht.nl](mailto:frutten@umcutrecht.nl))

Marc E.A. Spaanderman^5^ ([marc.spaanderman@mumc.nl](mailto:marc.spaanderman@mumc.nl))

Hester M. den Ruijter^4^ ([H.M.denRuijter-2@umcutrecht.nl](mailto:H.M.denRuijter-2@umcutrecht.nl))

Roel Bijkerk^1,2^ ([R.Bijkerk@lumc.nl](mailto:R.Bijkerk@lumc.nl))

Anton Jan van Zonneveld^1,2^ ([A.J.van_Zonneveld@lumc.nl](mailto:A.J.van_Zonneveld@lumc.nl))

*^1^Department of Internal Medicine (Nephrology) and ^2^Einthoven Laboratory for Vascular and Regenerative Medicine, Leiden University Medical Center, Leiden The Netherlands* *^3^Julius Center for Health Sciences and Primary Care, University Medical Center Utrecht, Utrecht University, Utrecht, The Netherlands .^4^Department of Cardiology, Division of Heart and Lungs, University Medical Center Utrecht, University of Utrecht, Utrecht, The Netherlands, ^5^Department of Obstetrics and Gynecology, Research School GROW, Maastricht University Medical Center, Maastricht, The Netherlands.*

| **Supplemental table 1.** Clinical characteristics of women with history of preeclampsia and asymptomatic LVDD compared to controls. | | |  |
| --- | --- | --- | --- |
|  | Controls  (n= 14) | Asymptomatic LVDD  (n=13) | |
| Age (years) | 46.8 (6.2) | 50.9 (8.6) | |
| Months post-partum | 217.2 (75) | 260.6 (86.5) | |
| Body weight (kg) | 76.9 (12.9) | 72.4 (13.4) | |
| Body surface area (BSA) (m^2^) | 1.9 (0.1) | 1.8 (0.1) | |
| BMI (kg/m^2^) | 27 (5.6) | 25.6 (5.3) | |
| Systolic blood pressure (mm Hg) | 120 (16.7) | 114 (10.8) | |
| Diastolic blood pressure (mm Hg) | 71.1 (6.2) | 69 (7.3) | |
| Total cholesterol | 5.4 (1) | 5.4 (1) | |
| HDL | 2 (0.6) | 1.5 (0.5) | |
| LDL | 3.0 (0.9) | 3.4 (0.9) | |
| Triglycerides | 1 (0.4) | 1.1 (0.5) | |
| **Echocardiography** | | |  |
| Left ventricular mass | 112.1 (25.6) | 131 (28.1) | |
| End diastolic volume | 110 (22.9) | 105.3 (19.2) | |
| End systolic volume | 42 (10.9) | 38.6 (8.5) | |
| Ejection fraction (%) | 61.9 (3.9) | 63.4 (3.6) | |
| Stroke volume | 76.6 (14.4) | 88.4 (23.7) | |
| Cardiac output | 4.9 (1.1) | 5.0 (1.4) | |
| EA ratio | 1.32 (0.3) | 1.24 (0.3) | |
| SD ratio | 1.17 (0.2) | 1.38 (0.4) | |
| E/E’ | 6.5 (1.6) | 9.2 (2.0)^1^ | |
| Left atrial volume index (mL/m^2^) | 29.9 (5.6) | 38.5 (9.5)^1^ | |
| Left ventricular mass index (g/m^2^) | 59.8 (12) | 72.3 (15.5) | |
| Relative wall thickness (RWT) | 0.30 (0.04) | 0.36 (0.06) | |
| Parametric data are presented as mean ± SD. ^1^p< 0.05 versus control according to an independent samples T-test. Controls were age, BMI and blood pressure matched. | | |  |

| **Supplemental Table 2.** (Sex-stratified) clinical characteristics of healthy controls and patients with LVDD. | | | | | | |
| --- | --- | --- | --- | --- | --- | --- |
|  | Total population | | Women | | Men | |
|  | No LVDD  (N= 244) | LVDD  (N= 122) | No LVDD  (N=160) | LVDD  (N=85) | No LVDD  (N= 84) | LVDD  (N=37) |
| Sex, male, n (%) | 84 (34%) | 37 (30%) |  |  |  |  |
| Age (years) | 61.7 ± 9.1 | 67.4 ± 9.4^1^ | 61.8 ± 8.6 | 67.1 (9.2) ^1^ | 61.5 ± 10 | 68.4 ± 9.9^1^ |
| BMI (kg/m^2^) | 27.1 ± 4.3 | 27.4 ± 4.8 | 27.1 ± 4.6 | 27.6 ± 5.3 | 26.9 ± 3.9 | 27.1 ± 3.4 |
| Systolic BP (mm Hg) | 144.4 ± 18.8 | 155.5 ± 19^1^ | 142.8 ± 18.2 | 154.5 ± 20^1^ | 147.4 ± 19.6 | 158.1 ± 16.4^1^ |
| Diastolic BP (mm Hg) | 86.1 ± 10.5 | 90 ± 10.9^1^ | 85 ± 10.1 | 88.8 ± 10.7^1^ | 88.1 ± 10.9 | 93.0 ± 10.7^1^ |
| Current or former smoker, n (%) | 163 (67%) | 74 (60%) | 108 (67%) | 47 (55%) | 55 (65%) | 27 (73%) |
| Hypertension, n (%) | 143 (59%) | 84 (70%)^1^ | 95 (60%) | 61 (73%)^1^ | 48 (58%) | 23 (64%) |
| Diabetes mellitus, n (%) | 17 (7%) | 12 (10%) | 7 (4%) | 10 (12%)^1^ | 10 (12%) | 2 (6%) |
| eGFR<60 ml/min | 19 (8%) | 18 (15%)^1^ | 15 (9%) | 11 (13%)^1^ | 4 (5%) | 7 (19%) |
| Creatinine (umol/L) | 68.8  (63.2-79.4) | 67.6  (60.8-76) | 65.3  (59.7-72.1) | 64.9  (59.6-70.6) | 81.1  (72.3-88.1) | 78.3  (69-89.5) |
| Total cholesterol (mmol/L) | 5.5 ± 1.1 | 5.3 ± 1.2 | 5.6 ± 1.1 | 5.5 ± 1.2 | 5.3 ± 1.1 | 5 ± 1.2 |
| HDL-cholesterol (mmol/L) | 1.4 ± 0.4 | 1.4 ± 0.4 | 1.5 ± 0.4 | 1.5 ± 0.4 | 1.2 ± 0.3 | 1.2 ± 0.2 |
| Triglycerides (mmol/L) | 1.9 ± 1.3 | 1.9 ± 1.2 | 1.7 ± 0.8 | 1.8 ± 1.0 | 2.2 ± 2 | 2 ± 1.6 |
| **Echocardiography** | | | | | | |
| Ejection fraction | 67.2  (63-73.4) | 66.9  (63-72.8) | 66.9  (62.5-73.4) | 66.9  (63.3-72.8) | 67.8  (63.9-73.6) | 65.3  (62-72.8) |
| EA ratio | 1.0 ± 0.3 | 0.9 ± 0.4^1^ | 1.0 ± 0.3 | 0.8 ± 0.3^1^ | 1.0 ± 0.3 | 1.0± 0.5 |
| E/E’ ratio | 8.6  (7.9-9.7) | 10.3  (9.1-12.8) ^1^ | 8.9  (7.9-10.0) | 10.5  (9.4-13) ^1^ | 8.5  (7.1-9.6) | 9.9  (8.3-11.7) ^1^ |
| LAVI (mL/m^2^) | 22.8  (18.8-27.4) | 30.9  (23.6-39) ^1^ | 22.1  (18.2-27.6) | 30.4  (21.1-38.3) ^1^ | 23.7  (19.7-26.6) | 32.2  (26.1-41.2) ^1^ |
| LVMI (g/m^2^) | 71.0 ± 15.5 | 86.4 ± 22.8^1^ | 68.9 ± 15.3 | 81.9 ± 20.1^1^ | 75.1 ± 15.2 | 97 ± 25.2 |
| RWT | 0.40  (0.36-0.46) | 0.45  (0.40-0.54) ^1^ | 0.39  (0.36-0.44) | 0.47  (0.39-0.55) ^1^ | 0.41  0.36-0.48) | 0.44  (0.41-0.51) ^1^ |
| BNP (pg/mL) | 17.9  (10-30.9) | 32.4  (15.3-66.6) ^1^ | 19.6  (10.5-32.9) | 28.1  (14.1-50.2) ^1^ | 16.4  (10-27.9) | 48.3  (16.4-86.6) ^1^ |
| e’ septal velocity (cm/sec) | 7  (6-9) | 6  (5-7) ^1^ | 7  (6-9) | 6  (5-7) ^1^ | 8  (6-9) | 6  (5-7) ^1^ |
| e’ lateral velocity (cm/sec) | 9  (8-11) | 7  (6-9) ^1^ | 9  (7-11) | 7  (6-9) ^1^ | 9  (8-11) | 8  (6-10) ^1^ |
| EA ratio, ratio of early (e) to late (a) ventricular filling; E/E’ ratio, ratio of mitral peak velocity of early filling (E) to early diastolic mitral annular velocity (E'); LAVI, left atrial volume index; LVMI, left ventricular mass index; RWT, relative wall thickness; BNP, brain natriuretic peptide; TPV, tricuspid valve regurgitation velocity. Parametric data is presented as mean ± SD or median ± SD. Non-parametric data is presented as median and IQR. Categorical data is presented as frequency and percentage. ^1^p<0.05 versus no LVDD as determined by Independent samples t-test for all normally distributed continuous variables, Pearson chi-square test for binary variables and Independent Samples Mann Whitney U test for non-normally distributed variables. | | | | | | |

| **Supplemental table 3.** Plasma profiling of X-chromosome located microRNAs in healthy women (n=3) and men (n=3). | | | | | |
| --- | --- | --- | --- | --- | --- |
| **Geometric mean of intensities in women (n=3)** | **Geometric mean of intensities in men (n=3)** | **Fold-change** | **MicroRNA ID** | **Parametric p-value** | **FDR** |
| 287,35 | 1176,27 | 0,24 | hsa-miR-664b-5p | 0,0044956 | 0,616 |
| 388,02 | 776,05 | 0,5 | hsa-miR-363 | 0,027357 | 0,957 |
| 16383,99 | 31288,25 | 0,52 | hsa-miR-660 | 0,0345057 | 0,957 |
| 131071,94 | 116771,88 | 1,12 | hsa-miR-130a | 0,0667662 | 0,957 |
| 37,62 | 1,41 | 26,6 | hsa-miR-888 | 0,0777908 | 0,957 |
| 39420,71 | 23712,06 | 1,66 | hsa-miR-27b | 0,0937884 | 0,957 |
| 741455,2 | 1001223,8 | 0,74 | hsa-miR-15b | 0,0994014 | 0,957 |
| 630,35 | 1231,9 | 0,51 | hsa-miR-503 | 0,1051315 | 0,957 |
| 3,25 | 31,27 | 0,1 | hsa-miR-18b | 0,1136259 | 0,957 |
| 488,88 | 1448,15 | 0,34 | hsa-miR-31 | 0,1213119 | 0,957 |
| 194130,37 | 64039,15 | 3,03 | hsa-miR-376c | 0,1241101 | 0,957 |
| 111498,63 | 154081,44 | 0,72 | hsa-miR-106b | 0,142438 | 0,957 |
| 1,32 | 34,3 | 0,038 | hsa-miR-708 | 0,1506042 | 0,957 |
| 4194304 | 5663777,2 | 0,74 | miR-20a | 0,1713483 | 0,957 |
| 2097151,1 | 3567956,1 | 0,59 | hsa-miR-451 | 0,180914 | 0,957 |
| 691,38 | 1149,4 | 0,6 | hsa-miR-214 | 0,1810847 | 0,957 |
| 228209,49 | 353986,23 | 0,64 | hsa-miR-103 | 0,1933647 | 0,957 |
| 1048576 | 1352002,9 | 0,78 | hsa-miR-491-5p | 0,2018449 | 0,957 |
| 99,27 | 1588,37 | 0,062 | hsa-miR-374c | 0,2042913 | 0,957 |
| 741455,2 | 1048576,5 | 0,71 | hsa-miR-26a | 0,2170785 | 0,957 |
| 38,5 | 261,98 | 0,15 | hsa-miR-93 | 0,2309528 | 0,957 |
| 108,89 | 1072,43 | 0,1 | hsa-miR-362-3p | 0,2357035 | 0,957 |
| 2642244,8 | 3329021,3 | 0,79 | hsa-miR-374b | 0,2415183 | 0,957 |
| 14937,66 | 19710,36 | 0,76 | hsa-miR-378 | 0,250814 | 0,957 |
| 122,22 | 13 | 9,4 | hsa-miR-513-5p | 0,2596716 | 0,957 |
| 1588,37 | 116,7 | 13,61 | hsa-miR-138 | 0,2660159 | 0,957 |
| 17158,86 | 26615,89 | 0,64 | hsa-let-7f | 0,2666379 | 0,957 |
| 11863278 | 26632170 | 0,45 | hsa-miR-19b | 0,2841158 | 0,957 |
| 1702,38 | 140,39 | 12,13 | hsa-miR-32 | 0,2912515 | 0,957 |
| 1,32 | 8 | 0,16 | hsa-miR-504 | 0,297337 | 0,957 |
| 111566783 | 143850974 | 0,78 | hsa-miR-223 | 0,2987522 | 0,957 |
| 13,61 | 222,86 | 0,061 | hsa-miR-501-3p | 0,304061 | 0,957 |
| 3565,77 | 11062,07 | 0,32 | hsa-miR-34a | 0,3057593 | 0,957 |
| 1,32 | 11,31 | 0,12 | hsa-miR-6857-5p | 0,3083428 | 0,957 |
| 88496,52 | 111498,63 | 0,79 | hsa-miR-28-5p | 0,3096702 | 0,957 |
| 3913423 | 5045851,8 | 0,78 | hsa-miR-20a | 0,3199371 | 0,957 |
| 322736,95 | 467087,51 | 0,69 | hsa-miR-29a | 0,32946 | 0,957 |
| 32767,99 | 45282,53 | 0,72 | hsa-miR-532 | 0,3304597 | 0,957 |
| 2144,86 | 198,55 | 10,8 | hsa-miR-545 | 0,3459876 | 0,957 |
| 630731,48 | 851708,44 | 0,74 | hsa-miR-374 | 0,3539606 | 0,957 |
| 35940,75 | 55749,33 | 0,64 | hsa-miR-98 | 0,3559323 | 0,957 |
| 2095,87 | 2702,35 | 0,78 | hsa-miR-502-3p | 0,3629847 | 0,957 |
| 2640,63 | 1097,5 | 2,41 | hsa-miR-193a-5p | 0,3902062 | 0,957 |
| 397335,93 | 287526,19 | 1,38 | hsa-miR-421 | 0,4154777 | 0,957 |
| 35119,87 | 43237,63 | 0,81 | hsa-miR-133b | 0,4232167 | 0,957 |
| 4,29 | 14,59 | 0,29 | hsa-miR-450b-5p | 0,4275385 | 0,957 |
| 588493,12 | 912838,35 | 0,64 | hsa-miR-125a-5p | 0,4403853 | 0,957 |
| 198667,97 | 233543,75 | 0,85 | hsa-miR-26b | 0,4498537 | 0,957 |
| 362259,98 | 445994,51 | 0,81 | hsa-miR-19a | 0,4503709 | 0,957 |
| 26007,97 | 13619 | 1,91 | hsa-miR-143 | 0,4517177 | 0,957 |
| 978355,74 | 1204497,6 | 0,81 | hsa-miR-20b | 0,4574599 | 0,957 |
| 31307384 | 40366832 | 0,78 | hsa-miR-188-5p | 0,4594748 | 0,957 |
| 776521,47 | 1176986,8 | 0,66 | hsa-miR-142-3p | 0,4671931 | 0,957 |
| 456419,17 | 676001,14 | 0,68 | hsa-miR-221 | 0,4950471 | 0,957 |
| 11856,03 | 17158,87 | 0,69 | hsa-miR-193b | 0,5027335 | 0,957 |
| 154081,37 | 108952,03 | 1,41 | hsa-miR-133a | 0,5085637 | 0,957 |
| 134,05 | 37,62 | 3,56 | hsa-miR-326 | 0,5114109 | 0,957 |
| 222,86 | 1072,43 | 0,21 | hsa-miR-500b | 0,5200279 | 0,957 |
| 84,45 | 268,11 | 0,31 | hsa-miR-548c-5p | 0,5244142 | 0,957 |
| 9855,18 | 8579,43 | 1,15 | hsa-miR-100 | 0,5316799 | 0,957 |
| 26615,88 | 29875,32 | 0,89 | hsa-miR-128 | 0,5568818 | 0,957 |
| 1448,15 | 2896,31 | 0,5 | hsa-miR-501-5p | 0,5581623 | 0,957 |
| 62576,47 | 73561,67 | 0,85 | hsa-miR-130b | 0,5652022 | 0,957 |
| 1482 | 1702,38 | 0,87 | hsa-miR-200a | 0,5978843 | 0,957 |
| 50827,91 | 53231,77 | 0,95 | hsa-miR-195 | 0,6086524 | 0,957 |
| 1912020 | 2353973,5 | 0,81 | hsa-miR-150 | 0,6123738 | 0,957 |
| 12706,97 | 15286,81 | 0,83 | hsa-miR-10a | 0,6135451 | 0,957 |
| 15653692 | 17570683 | 0,89 | hsa-miR-106a | 0,6163931 | 0,957 |
| 86475,29 | 106463,56 | 0,81 | hsa-miR-6086 | 0,6324049 | 0,957 |
| 58385,94 | 68635,45 | 0,85 | hsa-miR-340 | 0,6416747 | 0,957 |
| 978355,74 | 934175,01 | 1,05 | hsa-miR-331-3p | 0,6433317 | 0,957 |
| 19710,36 | 24266,3 | 0,81 | hsa-miR-142-5p | 0,6457907 | 0,957 |
| 274541,8 | 250305,95 | 1,1 | hsa-miR-21 | 0,652994 | 0,957 |
| 27874,66 | 25413,96 | 1,1 | hsa-miR-210 | 0,6529946 | 0,957 |
| 111,43 | 353,77 | 0,31 | hsa-miR-766-5p | 0,6584996 | 0,957 |
| 22655109 | 25429511 | 0,89 | hsa-miR-126 | 0,6612236 | 0,957 |
| 37,62 | 261,98 | 0,14 | hsa-miR-384 | 0,6636462 | 0,957 |
| 256156,6 | 308162,88 | 0,83 | hsa-miR-145 | 0,6645626 | 0,957 |
| 345900,99 | 308162,75 | 1,12 | hsa-miR-590-5p | 0,6692197 | 0,957 |
| 75281,07 | 82570,19 | 0,91 | hsa-miR-25 | 0,6701786 | 0,957 |
| 3106085,9 | 3406833,8 | 0,91 | mmu-miR-374-5p | 0,6701786 | 0,957 |
| 2352,53 | 2896,31 | 0,81 | hsa-miR-125b | 0,6756459 | 0,957 |
| 2767207,9 | 3651353,4 | 0,76 | hsa-miR-892b | 0,6772483 | 0,957 |
| 54476 | 77040,7 | 0,71 | hsa-miR-4767 | 0,6846429 | 0,957 |
| 3484,33 | 2765,52 | 1,26 | hsa-miR-365 | 0,6847668 | 0,957 |
| 1956711,5 | 1703416,9 | 1,15 | hsa-miR-146b-5p | 0,6873322 | 0,957 |
| 6658039,7 | 5534418,3 | 1,2 | hsa-miR-320 | 0,6880374 | 0,957 |
| 11585,23 | 7643,4 | 1,52 | hsa-miR-224 | 0,6883326 | 0,957 |
| 5042,77 | 6353,49 | 0,79 | hsa-miR-212 | 0,6901877 | 0,957 |
| 2702,35 | 3326,99 | 0,81 | hsa-miR-509-3p | 0,6941416 | 0,957 |
| 13,61 | 6,81 | 2 | hsa-miR-361-3p | 0,6975489 | 0,957 |
| 1703416,9 | 1449041,2 | 1,18 | hsa-miR-197 | 0,7085934 | 0,957 |
| 21137958 | 18401668 | 1,15 | hsa-miR-146a | 0,7125738 | 0,957 |
| 27874,66 | 33533,92 | 0,83 | hsa-miR-132 | 0,7177405 | 0,957 |
| 978355,74 | 871615,88 | 1,12 | hsa-miR-28-3p | 0,7224054 | 0,957 |
| 1,32 | 1,55 | 0,85 | hsa-miR-676 | 0,7235155 | 0,957 |
| 1783977,2 | 1415944,9 | 1,26 | hsa-miR-574-3p | 0,7237274 | 0,957 |
| 15653692 | 17169366 | 0,91 | hsa-miR-17 | 0,7280707 | 0,957 |
| 353986,07 | 406623,26 | 0,87 | hsa-miR-328 | 0,7384845 | 0,957 |
| 370727,44 | 445994,51 | 0,83 | hsa-miR-155 | 0,7412804 | 0,957 |
| 4002,45 | 3649,12 | 1,1 | hsa-miR-449a | 0,7470899 | 0,957 |
| 103,97 | 238,86 | 0,44 | hsa-miR-500 | 0,7542302 | 0,957 |
| 61147,23 | 53231,78 | 1,15 | hsa-miR-320d | 0,7598355 | 0,957 |
| 25,99 | 48,5 | 0,54 | hsa-miR-424 | 0,7646508 | 0,957 |
| 9195,21 | 6809,5 | 1,35 | hsa-miR-452 | 0,7681045 | 0,957 |
| 1589343,7 | 1415944,9 | 1,12 | hsa-miR-197 | 0,768971 | 0,957 |
| 233543,65 | 217904,07 | 1,07 | hsa-miR-27a | 0,7700844 | 0,957 |
| 831,75 | 3649,12 | 0,23 | hsa-miR-1298 | 0,7707723 | 0,957 |
| 287525,94 | 330280,74 | 0,87 | hsa-miR-486-3p | 0,7718609 | 0,957 |
| 61147,23 | 70239,75 | 0,87 | hsa-miR-628-5p | 0,7747372 | 0,957 |
| 15,63 | 10,56 | 1,48 | hsa-miR-1468 | 0,7909246 | 0,957 |
| 477,71 | 116,7 | 4,09 | hsa-miR-450a | 0,7943075 | 0,957 |
| 23712,06 | 21125,04 | 1,12 | hsa-miR-718 | 0,8094881 | 0,957 |
| 12133,15 | 13307,94 | 0,91 | hsa-miR-422a | 0,8138351 | 0,957 |
| 17559,93 | 18820,28 | 0,93 | hsa-miR-181a | 0,816752 | 0,957 |
| 6968,66 | 6353,49 | 1,1 | hsa-miR-99a | 0,8221977 | 0,957 |
| 18390,41 | 19710,36 | 0,93 | hsa-miR-885-5p | 0,823092 | 0,957 |
| 42250,08 | 46340,96 | 0,91 | hsa-miR-423-5p | 0,8246432 | 0,957 |
| 108951,98 | 101655,86 | 1,07 | hsa-miR-652 | 0,8468222 | 0,969 |
| 2353972,5 | 2196334,4 | 1,07 | hsa-miR-342-3p | 0,8489077 | 0,969 |
| 11068832 | 10327587 | 1,07 | hsa-miR-222 | 0,8576284 | 0,971 |
| 294246,56 | 274541,92 | 1,07 | hsa-miR-92a | 0,8823522 | 0,978 |
| 57052,37 | 58385,94 | 0,98 | hsa-miR-152 | 0,8899137 | 0,978 |
| 10321,27 | 9630,09 | 1,07 | hsa-miR-671-3p | 0,890179 | 0,978 |
| 24,25 | 40,32 | 0,6 | hsa-miR-513C | 0,9169698 | 0,978 |
| 24266,3 | 24833,5 | 0,98 | hsa-miR-345 | 0,9188487 | 0,978 |
| 10,08 | 7,64 | 1,32 | hsa-miR-508 | 0,9246038 | 0,978 |
| 891988,62 | 912838,35 | 0,98 | hsa-miR-140-5p | 0,9301869 | 0,978 |
| 9,4 | 12,41 | 0,76 | hsa-miR-23c | 0,9306357 | 0,978 |
| 1626492,3 | 1553042,9 | 1,05 | hsa-miR-513B | 0,9328287 | 0,978 |
| 3486463,5 | 3406833,8 | 1,02 | hsa-miR-92 | 0,9413313 | 0,978 |
| 4002,45 | 4191,74 | 0,95 | hsa-miR-1 | 0,9419048 | 0,978 |
| 416127,66 | 406623,26 | 1,02 | hsa-miR-139-5p | 0,9621393 | 0,991 |
| 3,4 | 3,56 | 0,95 | hsa-miR-6858-5p | 0,9786424 | 1 |

| Supplemental Table 4. Selection of plasma miRs involved in angiogenesis, X-chromosome origin, diabetes or cardiovascular disease. | | | | | |
| --- | --- | --- | --- | --- | --- |
| miR ID | Angiogenesis | X-linked | DM | DN | CVD |
| miR-20a | (1, 2) |  |  |  | (3) |
| miR-21 |  |  | (11) |  |  |
| miR-26b | (2) |  |  |  | (4) |
| miR-34a |  | (5) |  |  |  |
| miR-92a |  | (6) |  |  |  |
| miR-125a |  |  |  |  | (7) |
| miR-126 | (8, 9) |  | (8) | (8, 9) |  |
| miR-143 | (10) |  |  |  |  |
| miR-193a | (11) |  |  |  |  |
| miR-221 |  |  |  |  | (7) |
| miR-223 |  |  | (8) | (8) |  |
| miR-224 |  | (12) |  |  |  |
| miR-345 |  |  | (8) | (8) |  |
| miR-452 |  | (13) |  |  |  |
| miR-660 |  |  | (8) | (8) |  |
| References can be found below this table. X-linked, X-chromosome located; DM, Diabetes Mellitus; DN, Diabetic Nephropathy; CVD, cardiovascular disease | | | | | |

1. Deng HT, Liu HL, Zhai BB, Zhang K, Xu GC, Peng XM, et al. Vascular endothelial growth factor suppresses TNFSF15 production in endothelial cells by stimulating miR-31 and miR-20a expression via activation of Akt and Erk signals. FEBS Open Bio. 2017;7(1):108-17.

2. Stępień E, Durak-Kozica M, Kamińska A, Targosz-Korecka M, Libera M, Tylko G, et al. Circulating ectosomes: Determination of angiogenic microRNAs in type 2 diabetes. Theranostics. 2018;8(14):3874-90.

3. Gao G, Chen W, Liu M, Yan X, Yang P. Circulating MicroRNAs as Novel Potential Biomarkers for Left Ventricular Remodeling in Postinfarction Heart Failure. Dis Markers. 2019;2019:5093803.

4. Marketou ME, Kontaraki JE, Maragkoudakis S, Patrianakos A, Konstantinou J, Nakou H, et al. MicroRNAs in Peripheral Mononuclear Cells as Potential Biomarkers in Hypertensive Patients With Heart Failure With Preserved Ejection Fraction. Am J Hypertens. 2018;31(6):651-7.

5. Bernardo BC, Ooi JY, Matsumoto A, Tham YK, Singla S, Kiriazis H, et al. Sex differences in response to miRNA-34a therapy in mouse models of cardiac disease: identification of sex-, disease- and treatment-regulated miRNAs. J Physiol. 2016;594(20):5959-74.

6. Marzano F, Faienza MF, Caratozzolo MF, Brunetti G, Chiara M, Horner DS, et al. Pilot study on circulating miRNA signature in children with obesity born small for gestational age and appropriate for gestational age. Pediatr Obes. 2018;13(12):803-11.

7. Dudink E, Florijn B, Weijs B, Duijs J, Luermans J, Peeters F, et al. Vascular Calcification and not Arrhythmia in Idiopathic Atrial Fibrillation Associates with Sex Differences in Diabetic Microvascular Injury miRNA Profiles. Microrna. 2019;8(2):127-34.

8. Bijkerk R, Duijs JM, Khairoun M, Ter Horst CJ, van der Pol P, Mallat MJ, et al. Circulating microRNAs associate with diabetic nephropathy and systemic microvascular damage and normalize after simultaneous pancreas-kidney transplantation. Am J Transplant. 2015;15(4):1081-90.

9. Florijn BW, Duijs J, Levels JH, Dallinga-Thie GM, Wang Y, Boing AN, et al. Diabetic Nephropathy Alters the Distribution of Circulating Angiogenic miRNAs Between Extracellular Vesicles, HDL and Ago-2. Diabetes. 2019.

10. Climent M, Quintavalle M, Miragoli M, Chen J, Condorelli G, Elia L. TGFβ Triggers miR-143/145 Transfer From Smooth Muscle Cells to Endothelial Cells, Thereby Modulating Vessel Stabilization. Circ Res. 2015;116(11):1753-64.

11. Khoo CP, Roubelakis MG, Schrader JB, Tsaknakis G, Konietzny R, Kessler B, et al. miR-193a-3p interaction with HMGB1 downregulates human endothelial cell proliferation and migration. Sci Rep. 2017;7:44137.

12. Nair S, Jayabalan N, Guanzon D, Palma C, Scholz-Romero K, Elfeky O, et al. Human placental exosomes in gestational diabetes mellitus carry a specific set of miRNAs associated with skeletal muscle insulin sensitivity. Clin Sci (Lond). 2018;132(22):2451-67.

13. Tryggestad JB, Vishwanath A, Jiang S, Mallappa A, Teague AM, Takahashi Y, et al. Influence of gestational diabetes mellitus on human umbilical vein endothelial cell miRNA. Clin Sci (Lond). 2016;130(21):1955-67.

|  |  |  |  |  |  |
| --- | --- | --- | --- | --- | --- |

| **Supplemental Table 5.** Plasma miR levels in women with history of preeclampsia and asymptomatic LVDD compared to controls. | | | |
| --- | --- | --- | --- |
|  | Controls  (N= 14) | Asymptomatic LVDD  (N= 13) |  |
| miR-26a | 40.3 ± 23.1 | 34.8 ± 22.7 |  |
| miR-345 | 0.33 ± 0.13 | 0.37 ± 0.11 |  |
| miR-193a | 0.51 ± 0.29 | 0.95 ± 0.82 |  |
| miR-223 | 592.3 ± 205.5 | 614.0 ± 299 |  |
| miR-92a | 34.9 ± 18.5 | 38.3 ± 12.3 |  |
| miR-224 | 0.63 ± 0.30 | 0.95 ± 0.34^1^ |  |
| miR-143 | 0.15 ± 0.05 | 0.15 ± 0.10 |  |
| miR-221 | 4.03 ± 3.2 | 3.05 ± 1.97 |  |
| miR-660 | 0.60 ± 0.38 | 0.55 ± 0.26 |  |
| miR-34a | 0.06 ± 0.05 | 0.15 ± 0.21 |  |
| miR-26b | 3.33 ± 1.56 | 3.56 ± 1.90 |  |
| miR-125a | 0.76 ± 0.27 | 0.69 ± 0.38 |  |
| miR-452 | 0.05 ± 0.03 | 0.09 ± 0.05 |  |
| miR-126 | 51.32 ± 21.96 | 49.28 ± 21.92 |  |
| miR-21 | 2.86 ± 1.23 | 2.65 ± 1.20 |  |
| miR-20a  Ang-2 pg/mL  sFLT-1 pg/mL  sTrom pg/mL | 40.51 ± 18.88  1685 ± 409.6  89.28 ± 25.60  3589 ± 669.4 | 35.44 ± 17.85  2342 ± 699.3  103.3 ± 28.20  3648 ± 1014 |  |
| MiR expression values are presented as mean ± SD. ^1^p< 0.05 versus control according to an independent samples T-test. Controls were age-, BMI- and blood pressure- matched. Ang-2, Angiopoietin-2; sFlt-2, soluble fms-like tyrosine kinase-1; sTrom, soluble Trombomodulin   \| **Supplemental Figure 1 Ang-2 and miR expression profiling in plasma from women with a history of preeclampsia and asymptomatic LVDD (A-LVDD, n=13) compared to controls (n=14).** (A) Plasma miR-224 increased in women with a history of preeclampsia and A-LVDD (FC 1.5, p= 0.03). MiR-expression values are presented as mean ± standard error of the mean (SEM) and group differences are depicted as *p ≤ 0.05 an independent samples T-test. (B) Plasma Ang-2 levels increased in women with a history of preeclampsia and A-LVDD (fold change (FC) 1.4, p= 0.02). (C) Overview of miR association with echocardiography parameters (RWT and EF) and markers of microvascular injury (sTM and Ang-2). Correlations between variables were calculated using the Spearman rank correlation while p-values were adjusted with multiple testing through FDR with Benjamini Hochberg correction. \| \| --- \|   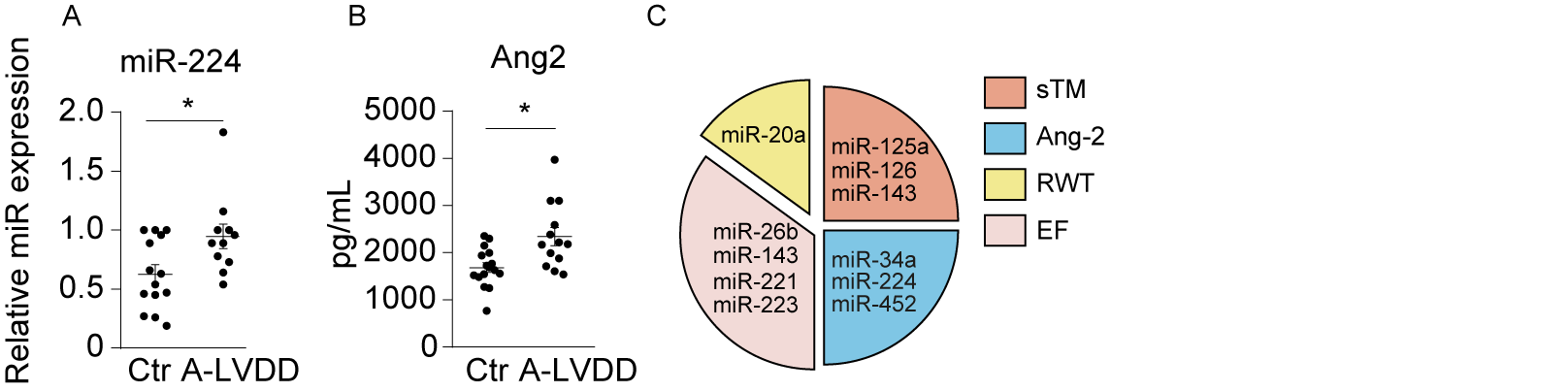 | | | |

| **Supplemental Table 6.** (Sex-stratified) clinical characteristics of patients with or without an eGFR < 60 ml/min with or without LVDD. | | | | | | |
| --- | --- | --- | --- | --- | --- | --- |
|  | Total population | | Women | | Men | |
|  | eGFR< 60 ml/min  Without LVDD  (N= 19) | eGFR< 60 ml/min  with LVDD  (N= 18) | eGFR< 60 ml/min  Without LVDD  (N=15) | eGFR< 60 ml/min  With LVDD  (N=11) | eGFR< 60 ml/min  Without LVDD  (N= 4) | eGFR< 60 ml/min  With LVDD  (N=7) |
| Sex, male, n (%) | 4 (21%) | 7 (39%) | 0 (0%) | 0 (0%) | 4 (100%) | 7 (100%) |
| Age (years) | 71.5 ± 9.1 | 76.9 ± 8.3 | 70.6 ± 9.2 | 76.3 (9.6) | 75 ± 8.8 | 77.9 ± 6.3 |
| BMI (kg/m^2^) | 27.4 ± 5.0 | 26.8 ± 6.8 | 27.4 ± 5.2 | 28.3 ± 8.2 | 27.5 ± 4.8 | 24.4 ± 2.3 |
| Systolic BP (mm Hg) | 150.8 ± 23.2 | 158.9 ± 14.7 | 149.6 ± 24.3 | 159.1 ± 13.9 | 155 ± 21.2 | 158.6 ± 17 |
| Diastolic BP (mm Hg) | 85.8 ± 12.7 | 88.9 ± 11.4 | 85 ± 13.4 | 88.6 ± 11.2 | 88.8 ± 11.1 | 89.3 ± 12.7 |
| Current or former smoker, n (%) | 9 (47%) | 11 (61%) | 5 (33%) | 6 (55%) | 4 (100%) | 5 (71%) |
| Hypertension, n (%) | 11 (61%) | 14 (82%) | 10 (71%) | 11 (100%) | 1 (25%) | 3 (50%) |
| Diabetes mellitus, n (%) | 2 (11%) | 3 (19%) | 1 (7%) | 2 (20%) | 1 (25%) | 1 (17%) |
| eGFR<60 ml/min | 19 (100%) | 18 (100%) | 1 (14%) | 2 (20%) | 1 (10%) | 1 (50%) |
| Creatinine (umol/L) | 97.3 (78.2-104.6) | 93.9 (81.3.8-104) | 87.6 (75.5-103.6) | 84 (73.9-100) | 107.9 (94-115.6) | 98.6 (93.8-122.1) |
| Total cholesterol (mmol/L) | 5.6 ± 1.0 | 5.2 ± 1.1 | 5.7 ± 1.0 | 5.4 ± 1.2 | 5.05 ± 0.8 | 4.8 ± 1.0 |
| HDL-cholesterol (mmol/L) | 1.4 ± 0.4 | 1.4 ± 0.3 | 1.5 ± 0.4 | 1.4 ± 0.4 | 1.1 ± 0.1 | 1.3 ± 0.2 |
| Triglycerides (mmol/L) | 2.0 ± 0.7 | 1.9 ± 1.3 | 2.0 ± 0.7 | 2.2 ± 1.5 | 2.1 ± 0.1 | 1.3 ± 0.3^1^ |
| **Echocardiography** | | | | | | |
| Ejection fraction | 66.5 (62.0-70.7) | 64 (57.3-74.5) | 68.3 (62.4-72.3) | 69.2 (63.5-81.7) | 63.7 (49.3-66.2) | 61.5 (36-64) |
| EA ratio | 0.8 ± 0.3 | 0.8 ± 0.3 | 0.8 ± 0.3 | 0.9 ± 0.3 | 0.8 ± 0.3 | 0.6 ± 0.1 |
| E/E’ ratio | 9.5 (8.4-11.6) | 12.5 (9.0-14.4) | 10.2 (8.9-14.5) | 12.8 (8.5-14.3) | 7 (6.2-8.1) | 12.2 (9.7-14.8)^1^ |
| LAVI (mL/m^2^) | 26.2 (20.3-30) | 28.9 (18.1-51) | 24.4 (20.1-27.2) | 28.9 (17-49.2) | 29.4 (21.6-33.5) | 30.1 (19.3-58.1) |
| LVMI (g/m^2^) | 73.1 ± 12.1 | 97.6 ± 28.7^1^ | 73.4 ± 12 | 90 ± 30 | 72 ± 14 | 109.6 ± 23.8^1^ |
| RWT | 0.41 (0.37-0.48) | 0.45 (0.41-0.58)^1^ | 0.41 (0.36-0.49) | 0.49 (0.42-0.61)^1^ | 0.40 (0.37-0.46) | 0.43 (0.41-0.52) |
| BNP (pg/mL) | 40.4 (11.9-45) | 87.5 (33.2-213)^1^ | 27 (11.4-45) | 83.6 (22.7-134.1)^1^ | 40.3 (25.5-49.2) | 211.1 (74.8-265.3) |
| e’ septal velocity (cm/sec) | 6 (5-9) | 5 (4-7) | 6 (4-7) | 5 (4-8) | 9.5 (6.5-11.8) | 5.5 (4-6.3)^1^ |
| e’ lateral velocity (cm/sec) | 7 (5.5-10.5) | 7 (6-10) | 7 (5-8.5) | 7 (7-10) | 11 (7-11) | 7.5 (5.3-9.8) |
| EA ratio, ratio of early (e) to late (a) ventricular filling; E/E’ ratio, ratio of mitral peak velocity of early filling (E) to early diastolic mitral annular velocity (E'); LAVI, left atrial volume index; LVMI, left ventricular mass index; RWT, relative wall thickness; BNP, brain natriuretic peptide; TPV, tricuspid valve regurgitation velocity. Parametric data is presented as mean ± SD or median ± SD. Non-parametric data is presented as median and IQR. Categorical data is presented as frequency and percentage. ^1^p<0.05 versus no LVDD as determined by Independent samples t-test for all normally distributed continuous variables, Pearson chi-square test for binary variables and Independent Samples Mann Whitney U test for non-normally distributed variables. | | | | | | |
